# Long-term outcomes of the observe-and-plan regimen in treating neovascular age-related macular degeneration: a retrospective real-life analysis

**DOI:** 10.1038/s41433-025-03830-1

**Published:** 2025-05-10

**Authors:** M. Sherif, Y. Derradji, A. Safi, I. Mantel

**Affiliations:** University Eye Hospital Jules-Gonin, Lausanne, Switzerland

**Keywords:** Diseases, Eye diseases

## Abstract

**Background/Objectives:**

This study aimed to evaluate the long-term (7 years) outcome of visual acuity in patients with neovascular age-related macular degeneration (nAMD) treated with anti-vascular endothelial growth factor (VEGF) agents following the observe-and-plan regimen.

**Subjects/Methods:**

A total of 195 eyes from 181 patients with nAMD (mean age 79.5 ± 6.9 years), with a mean follow-up duration of 66 ± 37 months, treated with intravitreal anti-VEGF (ranibizumab or aflibercept) were included in this retrospective study. The principles of the observe-and-plan regimen were followed, with follow-up exceeding 3 years in real-life settings. Data collected included visual acuity (VA), number of injections and visits, central retinal thickness, and any complications over 7 years from baseline.

**Results:**

The mean baseline VA was 63 ± 17 Early Treatment of Diabetic Retinopathy Study letters (Snellen equivalent 20/63), improving to 73 ± 14 at year 1. The initial visual gain was slightly reduced with a final mean VA of 70 ± 18 letters (Snellen equivalent 20/40) at year 7. The mean central macular thickness decreased significantly from 375 ± 129 at baseline to 276 ± 75 at year 1 and to 279 ± 87 at year 7. The mean annual number of injections decreased from 8.7 ± 3.2 in year 1 to 6.7 ± 3.7 in year 2 and to 5.5 ± 2.8 in year 7. The mean annual number of visits remained constant throughout, with 4.1 ± 1.3 visits in year 1 and 4.7 ± 1.7 in year 7.

**Conclusions:**

The observe-and-plan regimen was very efficient for treating nAMD in real-life settings, reducing the clinical burden on the medical system and patients, with excellent functional and structural long-term results.

## Introduction

Age-related macular degeneration (AMD) is a leading cause of irreversible blindness in patients >50 years of age. Neovascular AMD (nAMD) previously accounted for >80% of severe vision loss in patients with AMD [1, 2]. The introduction of intravitreal anti-vascular endothelial growth factor (VEGF) treatment was a major breakthrough, significantly improving nAMD prognosis [3, 4]. In 2006, the ANCHOR and MARINA trials demonstrated the safety and efficacy of ranibizumab in exudative AMD compared to verteporfin photodynamic therapy and sham treatment [5, 6]. In 2012, the VIEW 1 and 2 trials [7, 8] showed the non-inferiority of aflibercept compared to ranibizumab. However, the initial mean improvement in visual acuity (VA) may be lost over time due to factors such as undertreatment, atrophy, and fibrosis [9]. The effects of the treatment regimen and number of injections have been discussed in several previous studies [9–12]. A recent review concluded that the treat-and-extend regimen has substantial advantages over the Pro-Re-Nata and fixed-dosing regimens [10]. The treat-and-extend regimen predicts each subsequent treatment interval, leading to fewer injections than a fixed dosing regimen but better visual outcomes than Pro-Re-Nata. Undertreatment remains the main modifiable risk factor for avoidable visual loss over time [13, 14]. Therefore, adequate treatment intensity is crucial for long-term visual outcomes.

The observe-and-plan (OAP) regimen was developed to provide individually adjusted treatment frequency with anti-VEGF while reducing the clinical burden of monitoring visits [15–17]. It is based on the concept that adequate future treatment intervals can be accurately predicted after the initial loading dose [18]. By applying the predicted interval for up to 6 months ahead in a series of up to three injections, OAP reduces the number of monitoring visits needed [15]. However, the absence of monitoring visits during these injection series poses a risk of unrecognised recurrence, potentially leading to vision loss. The 2-year results of the study setting have been promising; however, the real-life and long-term results of the regimen have not yet been reported. Therefore, this study aimed to evaluate the long-term real-life outcomes of OAP, focusing on the number of injections and visits required to achieve these results.

## Subjects and methods

This retrospective study was conducted in the medical retinal department of a single tertiary referral centre (University Eye Hospital Jules Gonin in Lausanne, Switzerland) and included patients treated between 2011 and 2020. All applicable institutional and governmental regulations concerning the ethical use of human volunteers were followed. The study was approved by the local ethics committee under protocol number CER-VD 2017-00493. Patients gave written informed consent for retrospective analysis of their data.

### Patient selection

Our study involved a consecutive case series of patients with nAMD treated with intravitreal anti-VEGF (ranibizumab or aflibercept, or both) following the treatment protocol of the OAP regimen from baseline. The OAP regimen was our routine treatment regimen for all eyes with nAMD during the study period. No particular selection was performed. The follow-up duration was at least 3 years. The minimum age at AMD diagnosis was 50 years. Patients with confounding retinal pathologies, such as venous occlusions, diabetic retinopathy, or pathologic myopia, were excluded. Eyes receiving adjuvant treatments, such as photodynamic therapy with verteporfin for concomitant polypoidal choroidal vasculopathy, were also excluded. Image quality was not a selection criterion, as the study was intended to reflect real-life conditions. VA levels were also not a selection criterion.

### OAP regimen

This regimen, described previously [17, 18], consists of three initial loading doses of intravitreal anti-VEGF injections, followed by monthly observation visits until the disease recurrence interval is determined from the last loading dose injection. This interval was then shortened by 2 weeks and used for the next three injections without intermediate monitoring visits (up to 6 months). Subsequent treatment plans – again for a small series of typically three injections - were periodically adjusted for treatment interval, based on disease activity assessment, indicated by the presence (2 weeks shorter interval) or absence of any intra- and/or subretinal fluid (2 weeks longer interval). In case of haemorrhage, the interval was also shortened. If a 3-month interval was reached, and the disorder remained dry, the patient was offered more frequent observation or ongoing treatment every 3 months. There was no fluid tolerance criterion. Pigment epithelium detachment and vision were not considered as criteria for interval adjustment. Both ranibizumab and aflibercept were administered at the discretion of the treating physician. Furthermore, in cases of low vision levels (<0.1) over two consecutive visits, the physician discussed the option of ceasing treatment with the patient.

### Clinical routine investigations

VA was routinely measured using the Early Treatment of Diabetic Retinopathy Study (ETDRS) chart with automated refraction. The best available letter count was used for this study. All visits included a slit-lamp examination, intraocular pressure measurement, dilated fundus examination, and spectral-domain optical coherence tomography (OCT) on the Heidelberg Spectralis, acquiring a macular map of 6 × 6 mm with 49 lines (Heidelberg Engineering, Heidelberg, Germany). Additional examinations performed for all patients at baseline and thereafter, according to the treating ophthalmologist’s discretion, included fundus autofluorescence imaging, fluorescein angiography, and indocyanine green angiography (Heidelberg Angiograph; Heidelberg Engineering, Heidelberg, Germany).

### Collected data

Data collected at baseline and monitoring visits included best available VA, central retinal thickness (CRT), and number of injections received. For analysis, and as the visit dates were irregularly distributed, the last available data for each completed year of follow-up were transferred to the end of the year, including best available VA, CRT, number of injections, and visits performed during the corresponding year. The causes of interrupted follow-up were also evaluated. Cases of low vision (<0.1) were analysed for clinical cause. Severe adverse events were also recorded.

### Main outcome parameters

The primary outcome was the change in VA from baseline over 7 years. Additional outcomes linked to the primary outcome included the number of visits and injections performed per year of follow-up. Secondary outcomes included changes in CRT, the proportion of eyes with VA 20/40 or better, and those with VA 20/200 or worse at the end of follow-up.

### Statistical analysis

An Excel spreadsheet was used with monthly precision for longitudinal data, using the baseline data as a reference. The data were ordered according to the real-life appointments, even if the patient had postponed the appointment for any reason. The last available values were carried forward to the end of the corresponding follow-up year until the final observation. Only the yearly data were then used for evaluation.

In addition to descriptive statistics, paired or unpaired two-tailed t-tests (normal distribution checked) were used for follow-up evaluations as compared with baseline, with a *p*-value of <0.05 considered statistically significant.

## Results

This study included 195 eyes from 181 patients (mean age 79.5 ± 6.9 years; 67.9% female).

Data were collected over a mean follow-up period of 66 ± 19 months, spanning 3–7 years. Some patients were followed up for a longer period, but data after 7 years were excluded due to the small sample. The numbers of eyes with complete follow-up at 4, 5, 6, and 7 years were 175, 135, 107, and 52, respectively. At the last included follow-up, 107 eyes (54.8%) remained actively followed up, while 88 eyes (45.1%) were no longer followed up for the following reasons: 29 eyes (14.9%) continued the follow-up in a geographically closer healthcare facility, 23 eyes (11.8%) due to patient death, 18 eyes (9.3%) had no recurrence without treatment (>1 year) and continued follow up with their private ophthalmologist, six eyes (3.1%) due to low VA levels (counting fingers) and decided to stop the treatment, and 13 eyes (6.7%) for unspecified other reasons.

Baseline evaluation with angiography and OCT revealed type 1, 2, and 3 neovascularization in 119 (61.0%), 20 (10.3%), and 56 eyes (28.7%), respectively. In 30 eyes with type 1 neovascularization, an additional aneurysmal (polypoidal) choroidal neovascularization was found, representing 15.4% of the entire cohort. Serous pigment epithelium detachment (PED) was present in 34 (17.8%) eyes, fibrovascular PED in 73 (38.2%) eyes, and flat, irregular PED in 84 (44.0%) eyes. The angiographic lesion size at baseline was a mean of 2.0 ± 1.7 disc areas.

**Fig. 1:**
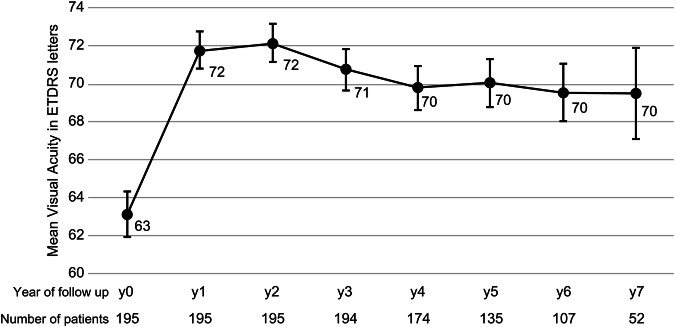
Mean visual acuity of eyes with neovascular age-related macular degeneration treated with ranibizumab or aflibercept following the observe-and-plan regimen. The number of eyes included in each year of follow-up is shown on the horizontal axis. The error bars represent standard error values.

Mean baseline VA was 63 ± 17 ETDRS letters (Snellen equivalent 20/63), which improved significantly to 72 ± 14 at year 1 (*n* = 195, *p* < 0.0001), 70 ± 15 letters (Snellen equivalent 20/40) at year five (*n* = 135, *p* < 0.0001), and 70 ± 18 letters (Snellen equivalent 20/40) at year 7 (unpaired t-test *p* = 0.01; paired test *n* = 52, *p* = 0.10). Figure 1 shows the mean VA values (±SE) over time, indicating good initial improvement with a mild decrease after year 2.

**Fig. 2:**
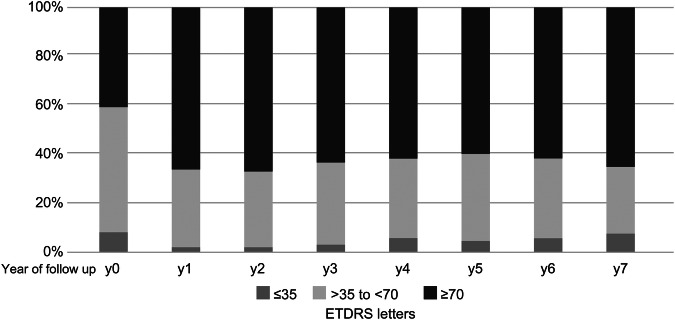
Visual Acuity change through the study period as per the initial VA at base line. Visual acuity groups and their relative distribution from baseline over 7 years of follow-up under the anti-VEGF retreatment regimen “observe-and-plan”: upper part (dark grey) = good vision with 70 ETDRS letters or better (Snellen equivalent of 20/40 or better); middle part (light grey) = reduced vision lower than 70 ETDRS letters but better than 35 ETDRS letters (Snellen equivalent between 20/40 and 20/200); lower part (mid grey) = low vision of 35 ETDRS letters or worse (Snellen equivalent 20/200 or worse). anti-VEGF anti-vascular endothelial growth factor, ETDRS Early Treatment of Diabetic Retinopathy Study.

The percentage of eyes with a VA of 20/40 (70 letters) or better increased from 41% at baseline to 66% at year 1 and remained nearly constant at 65% at year 7. The percentage of eyes with a VA of 20/200 (35 ETDRS letters) or worse decreased from 5.6% at baseline to 1.5% at year 1. However, at year 7, 5.7% had a letter count of 35 or worse. Across the entire cohort, 17 eyes (8.7%) had vision below 20/200 at the end of follow-up, with clinical reasons including fibrosis and atrophy in five eyes, atrophy alone in five eyes, fibrosis alone in four eyes, haemorrhage in one eye, and endophthalmitis in two eyes. Figure 2 shows the relative distribution of the VA groups by year.

**Fig. 3:**
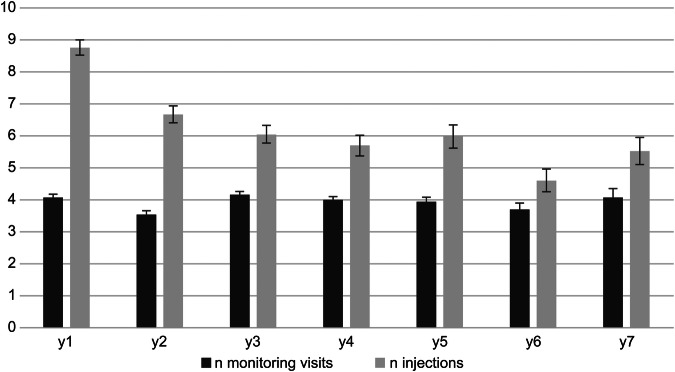
Number of monitoring visits and anti-VEGF injections (ranibizumab or aflibercept) per year of follow-up using the “observe-and-plan treatment regimen”. Anti-VEGF anti-vascular endothelial growth factor.

The mean CRT decreased significantly from 375 ± 129 µm at baseline to 276 ± 75 µm at year 1 (*p* < 0.0001) and then stabilised, with a final mean central macular thickness of 279 ± 87 µm at year 7 (*p* = 0.0009 compared with baseline). These results were achieved with a mean number of injections of 8.7 ± 3.2 in year 1, decreasing to 6.7 ± 3.7 in year 2 and then slowly decreasing over time to 5.5 ± 2.8 injections in year 7 (Fig. 3). The mean yearly number of monitoring visits was approximately four visits per year, remaining near constant throughout the study period, with 4.1 ± 1.3 visits in year 1, 3.5 ± 1.7 in year 2, and 4.7 ± 1.7 in year 7 (Fig. 3).

**Fig. 4:**
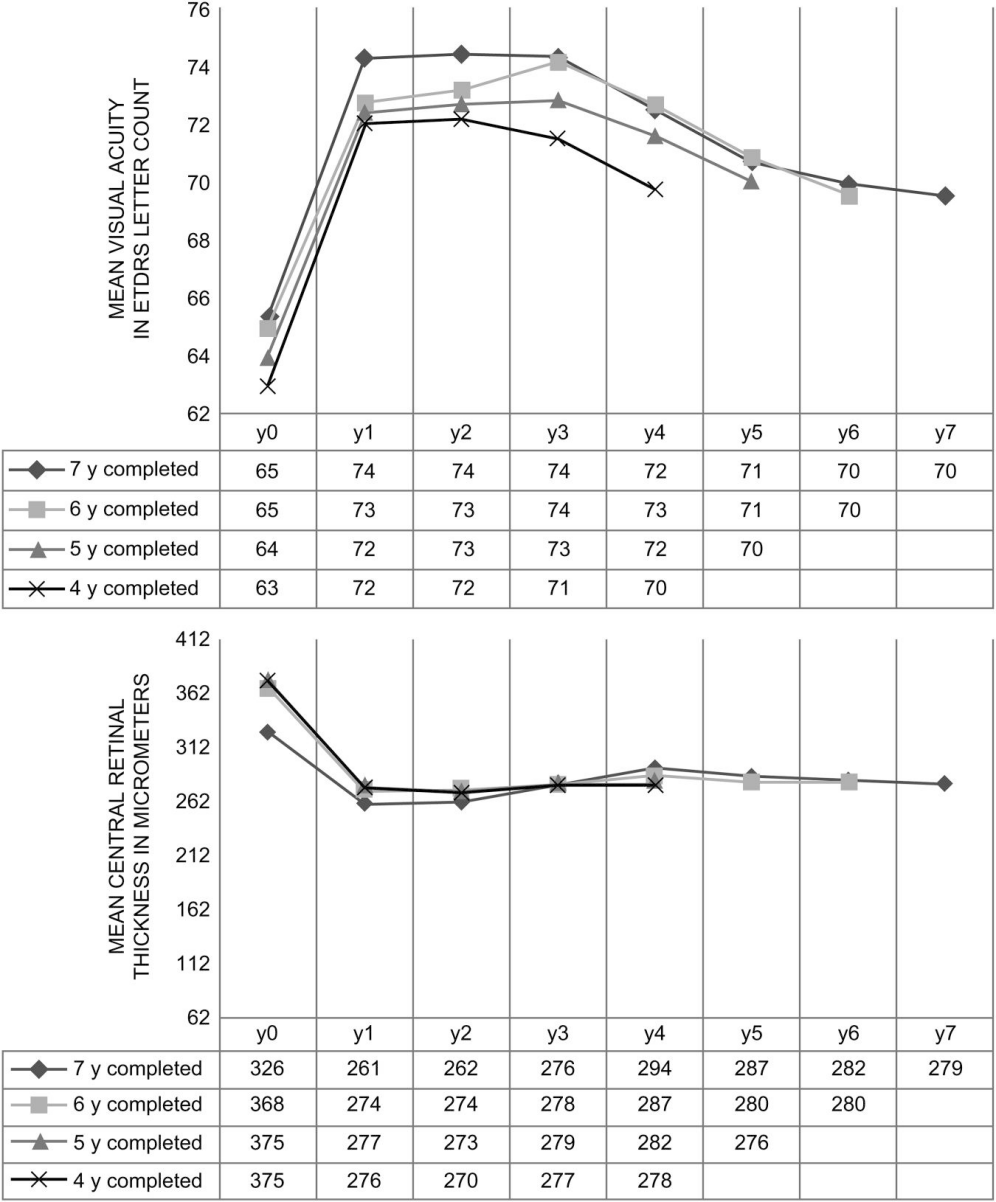
Subgroup analysis of mean visual acuity and central retinal thickness according to the number of completed years of follow-up (4, 5, 6, and 7 years).

Due to the decreasing number of patients included for follow-up, including some dropouts due to poor visual results (3.1%, six eyes), VA and CRT results were also analysed for subgroups with complete follow-up at 4, 5, 6, and 7 completed years (175, 135, 107, and 52 eyes, respectively) (Fig. 4). The VA outcomes differed only slightly among the groups, but the curves were parallel, starting from lower VA levels in the group with a shorter follow-up. The relative VA improvement was identical. The central retinal thickness curves were superposable.

Severe adverse effects were reported in 11 eyes, of which four presented with presumed endophthalmitis, four with non-infectious pseudo-endophthalmitis, and three with retinal pigment epithelial tears.

## Discussion

This study described the long-term outcomes of the OAP regimen in treating nAMD with ranibizumab or aflibercept in a real-life setting, showing good initial VA improvement (mean nine ETDRS letters) and excellent maintenance of functional improvement up to 7 years of follow-up (mean 7 ETDRS letters compared to baseline). Mild loss of mean VA over time (two ETDRS letters) was observed, and in 17 eyes (8.7%), the vision dropped below 35 ETDRS letters (Snellen 20/200), mainly due to fibrosis and atrophy (14 eyes, 7.2%). However, the proportion of eyes with VA of 70 ETDRS letters (Snellen 20/40) or better remained stable after initial improvement (41%, 66%, and 65% at baseline, year 1, and year 7, respectively).

The major advantage of the OAP regimen lies in the rapid determination of the optimal treatment interval (after loading dose), followed by a series of injections according to the individualised treatment need, thereby allowing a significantly reduced number of monitoring visits while maintaining the required treatment frequency for each patient. Hence, the number of visits was significantly lower than the number of injections (Fig. 3). This is more advantageous than a typical treat-and-extend regimen, where virtually every injection is preceded by a visit. The OAP regimen reduces the clinical burden for patients and health institutions. Additionally, the OAP regimen maintains the advantage of pre-planning, as in the treat-and-extend regimen, which is a major advantage of both regimens compared with the PRN regimen.

Previous long-term follow-up reports of anti-VEGF treatment in nAMD showed that the initial functional VA improvement was not maintained over time [19]. The initial improvement varied, but mostly depended on the baseline characteristics that determine potential visual recovery. However, once initial improvement has been achieved, the retreatment strategy and number of injections received have been identified as key factors for secondary degree visual loss [19, 20]. Early Pro-Re-Nata strategies, which react to multiple recurrences, have shown VA loss below the baseline level as early as 3–5 years after treatment initiation [21–24]. A review article linked this issue to undertreatment in real-life settings, showing that functional results were related to the mean number of injections administered [19]. With increasing awareness of the importance of frequent retreatment, more recent real-life data for long-term results, mostly using the proactive treat-and-extend regimen, have shown less rapid VA loss over time [25–27]. After 7–10 years, VA outcomes varied between −1 Snellen line (corresponding to −5 letters) [27] to +3 ETDRS letters [26] compared to baseline.

Thus, our real-life results of seven-letter improvement maintained through year 7 can be considered highly successful. This was achieved with a mean of 5.5–6.5 injections per year, after the initial year with a mean of 8.7 injections, similar to other long-term reports [23, 25, 26]. The difference between the OAP regimen and the treat-and-extend regimen lies mainly in the predictability of future treatment intervals [18], applying interval estimation to a short series of injections up to 6 months before the next monitoring visit [16]. The goal of applying a series of future treatment intervals is to reduce the clinical burden of monitoring visits while maintaining the individually required treatment frequency. However, with less frequent monitoring, there may be concerns that intermittent recurrences may remain unrecognised, leading to lower VA outcomes. Therefore, we evaluated our long-term clinical results, which were reassuring and did not indicate any signs that the regimen would be problematic in clinical settings. Patients were not undertreated.

Given the variability in the follow-up duration, we evaluated the impact of dropouts, which could potentially lead to better or worse results due to differing visual performances. Therefore, to maintain a stable number of analysed eyes per curve, we created groups according to the completed follow-up years. All groups reached a final mean VA of 70 letters (20/40), and the mean VA curves showed parallel behaviour. The VA changes from baseline were superposable. Therefore, our overall VA outcomes are not biased due to earlier dropout of poor outcomes and represent the OAP retreatment regimen potential to obtain sustained good VA outcomes in nAMD. However, the reasons for the 45.1% dropout rate were studied. The results showed that a large proportion of the dropouts were followed up at different institutions, usually the patient’s private ophthalmologist, due to a desire for a geographically closer provider (14.9%) or because active treatment was no longer needed, either because the patient was stable with good visual outcome (9.3%), or more rarely, due to poor visual outcomes (3.1%). The remaining 18.5% discontinued follow-up due to either death or unspecified reasons. These observations indicate that it is not likely that the results were overestimated due to the dropouts.

The number of injections per year was relatively high in our study. This is probably due to a strict no-fluid-tolerance strategy, in combination with the use of a very sensitive OCT device (Heidelberg Spectralis). Additionally, the mean number of approximately five injections for years 4, 5, 6, and 7 may appear relatively high. However, as the routine practice in our clinic was to send patients back to their private ophthalmologists when a stable situation was reached without treatment (1 year), the patients remaining in our institution mostly continued to present active nAMD, requiring ongoing treatment. This might explain the relatively high number of injections through to year 7.

The development of fibrosis and atrophy over time is partially unavoidable. It is typically associated with VA loss over time. This was observed in our cohort, although infrequently (7.2%).

Some limitations of this study should be acknowledged. First, the retrospective nature of the study, which is unavoidable when studying real-life results, also leads to irregular follow-up, including dropouts. Second, we did not differentiate between anti-VEGF agents used, as switching (only ranibizumab or aflibercept was used) was common. Finally, the number of eyes in this study was limited due to the monocentric design.

In conclusion, the OAP regimen applied in real-life settings proved to be both efficient and resource-sparing in treating nAMD, allowing for very good long-term VA outcomes while reducing the clinical burden on the medical system and patients. The number of injections required may be further reduced by the use of longer-acting anti-VEGF agents. However, this will also have to be demonstrated using real-life long-term studies. Further, prospective validation in larger cohorts is warranted.

## Summary

### What was known before

- The observe-and-plan (OAP) regimen was developed to provide individually adjusted treatment frequency with anti-vascular endothelial growth factor (anti-VEGF) agents while reducing the clinical burden of monitoring visits.
- By applying the predicted interval for up to 6 months ahead in a series of up to three injections, OAP reduces the number of monitoring visits needed.

### What this study adds

- The observe-and-plan regimen was very efficient for treating neovascular age-related macular degeneration in real-life settings, reducing the clinical burden on the medical system and patients, with excellent functional and structural long-term results
- The use of longer-acting anti-VEGF agents may further reduce the number of injections required.

## Data Availability

The datasets generated during and/or analysed during the current study are available from the corresponding author on reasonable request.
